# Highlights from the *e*cancer Future Horizons in Lung Cancer conference, 1–2 September 2016: Focusing on the future of treatment for NSCLC and SCLC

**DOI:** 10.3332/ecancer.2017.729

**Published:** 2017-03-23

**Authors:** Kayleigh Bassiri, Linda Cairns, Gordon McVie, Michael Seckl

**Affiliations:** 1 KB James Complete Academic Services, Plymouth, UK; 2 Istituto Europeo di Oncologia, via Ripamonti 435 Milano, Italy; 3 Cancer Studies, Kings College London, UK; 4 Faculty of Medicine, Department of Surgery and Cancer, Imperial College London, SW7 2AZ

**Keywords:** NSCLC, SCLC, biomarkers, targeted therapy, immune checkpoint inhibition

## Abstract

The ‘Future Horizons in Lung Cancer’ meeting was designed to bring leading scientists together alongside clinicians to discuss the most recent advances in lung cancer pathophysiology and treatment. The aim was to take those attending the event on a journey through decades of lung cancer research and understanding, with topics spanning from screening and surgical care to “omics” approaches for drug target and biomarker discovery. There were also several talks describing the role of radiotherapy in lung cancer and advancements in imaging techniques, aiding surgeons in their attempts to resect early lesions.

Current standards of care were both challenged and celebrated, while new and innovative immunotherapies also came into the spotlight. The meeting, held over two days, attracted a high calibre of speakers and delegates from across the globe. There were 10 sessions in total focusing on the latest therapeutic advances and predictions for the future of lung cancer treatment.

Highlights included a key note lecture from Dr Frances Shepherd packing 40 years of lung cancer research into a 40-minute presentation. Heated debates were had regarding the validity of maintenance therapy and immune checkpoint inhibitors that have taken the research community by storm. The latest developments in imaging, surgery, systemic and radiotherapy were presented over 10 sessions of exciting, innovative and stimulating presentations, leaving the audience lively yet pensive.

## Day one

Following a welcome and opening remarks from Professors Michael Seckl (Imperial College, London, UK) and Gordon McVie (King’s College, London, UK), day one of the meeting began with a talk from Professor Julian Downward (Francis Crick Institute, London, UK) kicking off the first session ‘NSCLC biology and new therapies’. Setting the scene, he shared some of the alarming statistics surrounding lung cancer before outlining the theme that would carry through the duration of his presentation; we need better therapies based on a deeper understanding of pathology. His talk focused on animal models of lung cancer and how they have progressed over the years. He talked about RAS mutations and their centrality to lung cancer, introducing the KRAS LA2 and KRAS LSL mouse models that are able to replicate the initiation and progression of human lung cancer *in vivo*. He showed data highlighting the powerful efficacy of combined therapeutic inhibition, including an almost complete reduction in tumour volume by targeting MAPK/ERK kinase 1 (MEK), mammalian target of rapamycin (mTOR) and the insulin growth factor receptor (IGFR). During his talk, Professor Downward was quick to acknowledge the limitations of mouse models, specifically their poor predictive power for clinical efficacy. He also highlighted the fact that mouse models have very limited mutational complexity which of course does not accurately reflect human lung cancer. He concluded by affirming that new developments are needed across all tumour models to make them more clinically relevant. During the question and answer session, there was interest from the audience as to whether any relevant models for brain metastases were either in use or under development, to which professor Downward answered no, stating that such a model was not likely to be translationally relevant.

The next speaker was Dr Rafael Rosell (Catalan Institute of Oncology, Barcelona, Spain) who discussed immunotherapies. He opened giving an overview of treatment with immune checkpoint inhibitors (ICI) and highlighted that for the first time, complete responses have been seen in patients taking ICI. He was cautiously optimistic stating that combination chemotherapy with nivolumab led to 2-year survival rate of 62% – but hastened to point out that different classes of non-small cell lung cancer (NSCLC) might respond differently to ICI. Following that, Dr Rosell described the case of a 62-year-old male who underwent third-line treatment with nivolumab and experienced complete remission measured by positron emission tomography-computed tomography (PET-CT) following six administrations. Dr Rosell pointed out that ICI causes upregulation of signal transducer and activator of transcription 3 (STAT3) and yes-associated protein 1 (YAP1), which correlate with decreased progression-free survival (PFS). He wrapped up his session by suggesting that a cotargeting approach combining ICI with STAT3/YAP1 inhibitors might be a powerful therapy in the future. Dr Rosell was asked about companion diagnostics for immunotherapy and how responses can be monitored – his reply was that while it is an important area, little is currently known.

Rounding off this first session was Professor Michael Seckl (Imperial College, London, UK), who concentrated on reversing resistance in lung cancer. He discussed functional genomic screens and their application in lung cancer research. Using these screens, we may be able to address three key questions; (1) can we sensitise cells to chemotherapy; (2) can we inhibit metastasis and (3) can we induce death in motile cells. He described the work of his group using the zebrafish as a model and reported the identification of a novel target, ‘Kinase X’. He showed that real-life patients with elevated levels of Kinase X had decreased overall survival than those without. Professor Seckl identified several inhibitors on the market that have shown efficacy pre-clinically and could potentially be repurposed in this setting, leading him to call for a proof of concept trial and identification of a companion diagnostic biomarker. The conclusion of the talk garnered interest from the audience about resistance to kinase x inhibition – a question he had prepared to answer. Prof Seckl readily acknowledged that this would likely become an important issue in the future, further highlighting the importance of companion diagnostics and biomarkers.

The second session ‘Screening, surgical care and new developments’, saw Dr Claudia Henschke (Mount Sinai Hospital, New York, USA) take to the podium. She began with an introduction to the early lung cancer action programme (ELCAP) – the largest CT screening cohort in the world with over 66,000 participants in 10 countries. She talked about how to avoid false positives during screening and stressed the importance of balance between finding disease early, and unnecessary work-up. She highlighted that at baseline for every 1000 patients screened, 20–30 cancers will be identified and 80% of those will be stage 1. At annual repeat screening, this figure changes to 5–9 cancers per 1000 patients screened, again with 80% at stage 1. At this point, she stressed that there should be a profound difference between the regimens of screening. She also outlined the differences between nodules; that sub-nodules are generally less aggressive than solid nodules. The ELCAP initiative found that no metastasis occurred when the solid nodule component was < 10 mm. Her final comments focused on the importance of integrating advances in lung cancer across screening, radiotherapy and systemic therapy in order for lung cancer research to progress.

Up next in session two was Dr Francesco Di Meco (Istituto Nazionale Neurologico Carlo Besta, Milan, Italy) discussing ‘Future smart surgical approaches for brain metastases.’ A series of slides were presented with the overall conclusion that whole brain radiotherapy only modestly improves PFS and does not improve overall survival with functional independence. He further pointed out that studies are required to decipher whether aggressive surgical interventions improve quality of life or not. The main body of the talk focused on different surgical methods – with stereotactic radiosurgery (SRS) being earmarked as promising for two reasons; it provides high-dose radiation to a defined and localised area and is not limited by tumour location or depth. He went on to describe how to measure the effectiveness of treatment identifying overall survival, local control, cumulative morbidity and ease of follow-up as the primary criteria. He then concentrated on tools for safe maximal resection such as image-guided surgery, pre-surgical MRI mapping and surgical simulation and how these have become more and more advanced and likely to be used more extensively in the future.

Professor John Field (University of Liverpool, UK) was the next speaker and talked about how best to organise a national screening programme. He opened his presentation with a stark fact – that 34% of patients are diagnosed at emergency presentation with the least possible chance of successful treatment. He reiterated the importance of screening in the future of lung cancer research and the fact that early stage disease amenable to surgery has a relatively good outlook. The necessity for high-risk stratification and identifying those patients most likely to benefit from lung cancer screening was discussed and much of his talk centred on the most ‘hard to reach’ patients, which generally include smokers and those from lower socioeconomic backgrounds. These patients are reluctant to take part believing that the damage has already been done and that screening will be of no benefit to them. He gave a brief overview of ACE, a programme to ‘accelerate, coordinate, and evaluate learning to achieve the earlier diagnosis of cancer’, and concluded by making a plea for recruitment suggesting ways to encourage patients to take part. These include ‘healthy lung events’, that show people how big the difference can be between ‘real age’ and ‘lung age’, depending on various lifestyle factors.

Last to speak in this session was Dr Ugo Pastorino (Istituto Nazionale Tumori, Milan, Italy) talking about ‘current state of the art in surgery’. He first outlined four important considerations for lung cancer surgery; access to the chest, resection volume, early detection, and multimodal therapy. He talked about minimally invasive surgery, and its advancements over the last 15 years. He then touched on the use of robots in surgery and stated that in his opinion they are still yet to prove their benefit. Dr Pastorino talked about intraoperative mediastinal staging and showed data highlighting an increased survival benefit proportional to the number of nodes evaluated. Echoing Dr Henschke, he stated that non-solid lesions do not require immediate treatment and active surveillance is perfectly adequate. The conclusion was that modern surgery is safer for high-risk individuals but more complex in higher stage disease, and overall, it is more cost-effective.

The concluding panel discussion attracted a question about patient selection being highly focused on smokers. The questioner made a point that women of menopausal age who are never-smokers can also be considered higher risk than the general population. Dr Henschke responded stating that 30,000 people die each year from lung cancer that have never smoked. Diagnosed emphysema in these patients is just as high a risk factor for lung cancer as smoking and should not be ignored.

The third session of the day was entitled ‘Radiotherapy - What is new?’ It was opened with an energetic talk from Professor Corinne Faivre-Finn (The Christie NHS Foundation Trust, Manchester, UK). She presented European organisation for research and treatment of cancer (EORTC) guidelines calling for the implementation of stereotactic body radiotherapy (SBRT) into routine clinical practice stating that it can induce local control of 90%, compared with conventional radiotherapy (RT) that is only around 50%. She acknowledged that in order to consistently achieve this we need accurate staging, assessment of respiratory motion, highly planned and image-guided techniques. She described the RTOG0617 trial as a pivotal study for defining the standard of care in NSCLC [1]. Both arms included doublet chemotherapy, and the trial showed that a dose of 74 Gy radiation was not better than 60 Gy and was in fact potentially harmful. Promisingly, the overall survival (OS) for both arms was the best ever reported in this patient population. She followed this with a meta-analysis pitting concurrent treatment against sequential treatment, showing that local control was better with concurrent treatment. The talk ended with data from the CONVERT trial which assessed high-dose RT once a day versus twice a day to address concerns over potential toxicity with twice a day therapy [2]. Both yielded similar survival rates with no significant increase in toxicity with the twice a day regimen. As such, either could be considered the standard of care depending on patient choice and departmental logistics.

The second and final talk of this session came from Dr David Landau (Guy’s and St Thomas’ NHS Foundation Trust, London, UK) talking about future directions in radiation medicine. He outlined the SABRTooth trial for early operable NSCLC which is planned to pit SABR against surgery for the treatment of residual disease. He moved on discuss the difference between an ‘oligometastatic state’, and a ‘non-oligometastatic state’, the former being more amenable to ablative RT and potential cure than the latter. He described the results of a trial looking at local consolidative therapy (LCT) versus no LCT after first-line systemic therapy, showing that patients receiving LCT experienced PFS of 11.9 months versus 3.9 months. He believes a phase-III trial is now justified to ascertain whether OS will also improve with LCT. The session concluded with the combination of RT plus immunotherapy, and the synergistic effects that the two could have in NSCLC adding that several trials are ongoing.

Both speakers had covered proton therapy during their presentations and described the results of a study that found no significant advantage over photon therapy [3]. The ensuing question and answer session prompted Michael Seckl to describe it as ‘all the rage’, but then questioned how applicable it would be in routine daily practice. Professor Faivre-Finn jumped to the defence of proton therapy saying that in the trial, maturing proton therapy had been tested against sophisticated photon therapy making the comparison flawed. Both agreed more trials were needed and that the story is far from over. They were also asked about priming the immune system with radiotherapy and agreed this occurs primarily through immunogenic cell death – with Professor Faivre-Finn suggesting that the best method going forward may be to administer both forms of therapy concurrently.

The fourth session of day one was all about ‘Imaging and novel PET tracers’. Dr Giulia Veronesi (Humanitas Research Hospital, Milan, Italy) was first up discussing the current state of the art. Dr Veronesi used the results of the COSMOS trial to highlight the usefulness of CT scanning, with almost 80% of tumours detected at stage I [4]. Delayed diagnosis was highlighted as an issue with 16/190 (8%) stage II–IV cancers not recognised one year before, mainly due to location. The talk also covered the use of imaging during surgery to identify distant unexpected metastases and gave an overview of radiomic nodule classification using a non-surgical approach – showing a significant prognostic advantage over surgery. Dr Veronesi also described how micro-RNA (miRNA) can improve the sensitivity of PET diagnosis – where PET plus miRNA screening correctly classified 93% of tumours.

The last talk of this session came from Professor Tony Ng (King’s College, London, UK) who focused on the future of imaging in NSCLC. The first point made was that not all metastases respond to chemotherapy in the same way – therefore whole body imaging is useful to monitor response to systemic therapy and how resistance evolves. Part of Professor Ng’s presentation focused on circulating exosomes and how they too can be used to monitor tumour evolution. Using the example of epidermal growth factor receptor (EGFR) inhibition resulting in ‘rewiring’ of signalling to Erb-B2 Receptor Tyrosine Kinase 3 (HER3), he reported on patients treated with the third-generation EGFR inhibitor osimertinib who eventually relapsed due to increased HER3 expression. A HER3 radiotracer was able to monitor the development of resistance through circulating exosomes in the blood, rather than having to wait for measurable tumour growth. A cMET PET tracer is also under development that can be used to track secondary resistance.

The work of Dr Ng attracted several questions from the audience. He was asked whether any agents had been developed to detect PD-L1 in circulating cells. He replied that they had recently made a PD-L1 PET imaging agent that could be used to identify patients likely to respond to immune checkpoint therapy. The final question of the session related to exosomes in early disease – do they hold positive predictive value? The answer is yes and this is something that should be looked at.

Session 5 saw Dr Frances Shepherd take the audience on a whistle-stop tour through 40 years of lung cancer research. In an entertaining and energetic presentation, Dr Shepherd highlighted just how far we have come, despite prognoses at times still looking bleak. She talked about targeted therapies in lung cancer while making the point that what might be statistically significant in a trial setting does not necessarily translate into the clinic. She also said that her personal choice is to treat patients with the well-tolerated combination of pemetrexed plus carboplatin which, in her own words, ‘is based on no science, just 40 years of experience’. She went on to discuss maintenance therapy with pemetrexed showing that some patients now experience 2-year OS. Results of the BR-10 trial were presented, showing that adjuvant chemotherapy led to a 20% difference in OS at 5 years compared with observation alone. Moving on to second line, she asked, ‘can we do better adding targeting agents?’ Sadly, no improvement is seen by adding bevacizumab or erlotinib (at least not in patients without EGFR driver mutations). For those with driver mutations – first-line anaplastic lymphoma receptor tyrosine kinase (ALK) or EGFR inhibition is now first choice and rightly so. Naturally, the following slides focused on EGFR tyrosine kinase inhibitor (TKI) resistance, and osimertinib or as Dr Shepherd described it ‘the jewel of the drugs’, with almost no native EGFR toxicity and a high response rate. The talk ended with immune checkpoint inhibitors. Dr Shepherd’s parting words of wisdom were ‘the immunotherapy tsunami is upon us, but boy will it bankrupt us!’ Combination immunotherapy is a revelation but could prove extremely costly; the notion of cost in today’s clinic, being a pertinent theme in her presentation.

Session 6 was all about controversies in NSCLC. Dr’s Benjamin Besse (Institut Gustave Roussy, Paris, France) and Jaishree Bhosle (The Royal Marsden NHS Foundation Trust, London, UK) went head-to-head debating the pros and cons of maintenance therapy. Dr Besse vehemently outlined his clear reasons against it the with opening statement, ‘maintenance is a lie!’ Highly selected patient populations (only those without progressive disease) are eligible. The control arm is always poor and badly designed switch maintenance trials often have undertreated control arms. In the AVAPERL trial, patients were randomised after surviving four cycles of chemotherapy with little toxicity, making the maintenance trial data look much better than it is in real life [5]! Overall, he made a pretty convincing case against maintenance therapy – ending on the suggestion that the point of maintenance is simply to sell more drugs. Dr Bhosle approached the debate from the opposite angle but did acknowledge that maintenance trials can be confusing, with different numbers of chemotherapy cycles between arms and differences in the treatments patients received prior to progression. She went on to show a three-month OS advantage for patients switched to pemetrexed maintenance – and a case in point was that OS is ultimately what matters. Many patients say while their quality of life may be compromised, more time with their families is more important. Maintenance should be considered when patients have a good performance status, when there is a low threshold for discontinuation and when patients are able to attend the appointments. While we wait for immunotherapies to really take-off, maintenance therapy is ready and waiting for those fit enough to take it. The audience were decidedly on the side of Dr Bhosle with the majority holding up their green cards in favour of maintenance therapy.

Dr Mary O’Brien was up next to ponder the hot topic of PD-L1 and other biomarkers for selecting patients for immune checkpoint therapies, giving her arguments in favour. She stressed the importance of testing all patients to find the right target and prescribe the most appropriate drug quickly. In the case of crizotinib – for patients with the driver mutation, an objective response rate (ORR) of 74% was reported compared with 45% for patients receiving standard chemotherapy; a true testament to getting it right first time. She outlined approved second-line agents (atezolizumab, nivolumab and pembrolizumab) and the possibility of first-line treatment with these inhibitors. Patients were required to be > 5% PD-L1 positive for the first-line assessment of nivolumab; however, no significant advantage was noted compared with control. The pembrolizumab study required > 50% positivity and results are reported to show a significant survival advantage. This is pretty convincing evidence for PD-L1 expression as a biomarker to predict response to these drugs.

Professor Gordon McVie (King’s College, London, UK) drew the proceedings of day one to a close, arguing against PD-L1 as a biomarker. He was quick to address the previous talk stating his reasons for perhaps being more cautious about immune checkpoint inhibitors. He made valid points regarding the fact that different trials use different thresholds for PD-L1 positivity and that trial data is anything but gathered uniformly; from the timing of biopsies to the method of tissue fixation and time in transit – these can all affect PD-L1 staining and limit the quality control of immunohistochemistry data. Professor McVie seemed angered almost at the lack of multivariate analysis and lack of correlation with toxicity ending with the notion that in his opinion – they are yet to live up to their reputation!

Day one had certainly whet our appetites for what the second day of the conference had in store.

## Day two

Day two began with a session focussing on advances in small-cell lung cancer (SCLC). Dr Marianne Nicolson (Aberdeen Royal Infirmary, Scotland, UK) started proceedings discussing current standards of care. She began with an analogy likening SCLC to a brick wall – but optimistically added that we are indeed starting to break it down, primarily in less advanced, limited disease (LD). She believes that PET imaging should be more widely used having been proven beneficial through randomised controlled trials (RCTs). She drew several comparisons with NSCLC and agreed that combination chemotherapy is better than single agent therapy, with maintenance therapy yet to be properly addressed in SCLC. Dr Nicolson reiterated the survival advantages of concurrent RT in LD SCLC and presented results from the CONVERT trial echoing the conclusions drawn by Professor Faivre-Finn the previous day [2]. Touching briefly on extensive disease, she outlined the benefits of prophylactic cranial irradiation and thoracic consolidation therapy in these patients. She finished with an overview of SCLC RCTs concluding that unfortunately we still have a long way to go. On a more positive note, she stressed that this could be accomplished with better understanding of the molecular biology of SCLC fostering a more intelligent approach to treatment.

Finishing up this first session was Dr Fiona Blackhall (The Christie NHS Foundation Trust, Manchester, UK). Her talk, ‘New therapies’, began with a description of SCLC as being ‘fast, hungry and unstable’. Dr Blackhall was quick to highlight the shortcomings of SCLC research that over 60 agents have been studied and yet none have been licensed. Antiangiogenics have been extensively studied and shown no real efficacy, so the focus has shifted to other signalling pathways. Targeting IGFR, mTOR, EGFR and hepatocyte growth factor (HGF) have all been negative so far, but we await results of V-Akt murine thymoma viral oncogene homolog (AKT)/phosphoinositide 3-kinase (PI3K) studies in SCLC. Dr Blackhall highlighted known driver mutations with most falling below 10% incidence but showed that Notch gene family mutations occur in 25% of patients. One particular drug that looked set to succeed was oblimersen – targeted against B-cell lymphoma 2 (BCL-2) (overexpressed in 75% of SCLC). Unexpectedly and very disappointingly, results showed that standard chemotherapy gave a significant survival advantage over oblimersen. Targeting stem cell pathways has shown the most promise – with a notch 2/3 targeted agent, tarextumab, inducing an ORR of 84% in a phase-IB trial. Antibody–drug conjugates and targeting the DNA damage response were also presented with both having shown promising results. Dr Blackhall concluded by emphasising the difficulties in obtaining tissue biopsies, and placed her hopes on circulating tumour cell (CTC) analysis in the future suggesting that better CTC/PDX models would greatly improve our progress.

During the question and answer session, Dr Blackhall said she had been approached frequently in the last year by pharmaceutical companies to carry out feasibility studies for new agents in small-cell – providing new hope for the future of SCLC treatment.

The second session of the day was all about ‘Omics, early detection and novel therapies.’ First to speak was Dr Olivier Pardo (Imperial College, London, UK) introducing us to the world of metabolomics. The presentation focused largely on EGFR inhibition and the now notorious T790M-mediated resistance to therapy. Dr Pardo offered a very comprehensive account of what may be to blame: glutathione. He stated that it is downregulated in resistant cancer cells compared with sensitive cells and after performing several experiments to confirm the link between glutathione and T790M mediated resistance, Dr Pardo sought to test whether glutathione would be relevant in the clinic. His team found that after T790M resistance had occurred in patients – enzymes responsible for the synthesis of glutathione decreased. The talk concluded with the suggestion that GSH inhibition may be a good delay tactic before introducing more expensive third-generation compounds to combat resistance, and that glutathione catabolic enzymes could be good predictors for the development of resistance to EGFR inhibition.

Dr Adele Murrell (University of Bath, UK) followed with a talk on epigenetics – in her opinion widely regarded as too difficult to understand by many researchers. She took the opportunity to dispel some of the myths, explaining that epigenetics is simply the way DNA responds to its environment at any given time or under any given circumstances. Dr Murrell likened DNA to musical notes, and epigenetics correspond to how loud or fast the music will play. Perhaps the most common epigenetic mechanism that most will know is methylation. In lung cancer, cyclin-dependent kinase inhibitor 2A (CDKN2A) methylation has been identified as an early marker of disease in smokers. An interesting idea was the use of cancer detection dogs to analyse exhaled breath condensates to identify early disease, met with intrigue from the audience. During the question and answer session, this proved to be a popular area for discussion leading to the simple conclusion that we need more researchers working in the early detection space. Dr Murrell noted that overall epigenome inhibitors tend to be quite non-specific, while some histone deacetylase (HDAC) inhibitors do have efficacy they are best used in combination with other chemotherapeutics.

The final session of the conference shone the spotlight on immunotherapies in both NSCLC and SCLC, consolidating much of what had been presented over the course of the meeting. Opening the session was Professor Giuseppe Giaccone (Georgetown University, Washington D.C., USA) talking within the context of NSCLC. He was followed by Professor Dean Fennell (University of Leicester, UK), representing the SCLC situation. Professor Giaccone presented nivolumab data that left the audience in no doubt of its value – with a 1-year survival rate almost double that of docetaxel as reported by the Checkmate 017 trial in the second-line treatment of NSCLC [6]. He also confirmed that retrospective PD-L1 expression analysis of NSCLC patient samples before the initiation of nivolumab therapy identified proportionally increased OS for PD-L1-positive patients. Professor Fennell showed that PD-L1 expression is significantly lower in extensive SCLC compared with LD, demonstrating that it is not a good target in more advanced disease. Several other immune checkpoint inhibitors were introduced highlighting the potential of the market to become more convoluted over the coming years. The combination of nivolumab and ipilimumab was presented by both speakers, echoing the key note from Dr Frances Shepherd from the previous day. Indeed, the data presented would place the combination at the forefront of immunotherapy for NSCLC with efficacy across all PD-L1 expression levels and a similar adverse event profile to that seen with single agent therapy [7, 8]. The success of the combination was nicely demonstrated with a case study featuring the response of a 52-year old former smoker to nivolumab plus ipilimumab, experiencing a 53% reduction in tumour size. The combination was also assessed in SCLC and led to a survival advantage over nivolumab alone. Interestingly, tumours with high non-synonymous mutational burden respond much more favourably to PD-L1 inhibition. Regarding the future, focal adhesion kinase (FAK) inhibition in combination with PD-L1 inhibition may be a powerful approach. FAK inhibition has failed previously and there is a link between this phenomenon and PD-L1 expression.

Overall, we are slowly but surely moving in the right direction. Immune checkpoint inhibition may prove to be our best option particularly in SCLC, considering our knowledge of mutational changes are yet to translate into effective therapy.

## Conclusion

The meeting was brought to a close by Professor Michael Seckl who offered his thanks to the speakers and participants after two days of presentations that certainly did not disappoint. Meetings like this never fail to stimulate discussion, collaboration and encourage us to really think about our research in the wider context. The take-home messages were that immune checkpoint inhibition is set to take off, and personalised medicine is becoming more realistic in a world where sophisticated approaches to research have identified a wealth of targets and biomarkers. Our imaging techniques are advancing, our screening programmes are identifying disease earlier and we can finally offer patients more hope for the future. We have more information than ever before – and the challenge now is to decipher just how to translate this information into relevant clinical outcomes for the people who matter the most: our patients.
